# Can microbial‐based insecticides replace chemical pesticides in agricultural production?

**DOI:** 10.1111/1751-7915.14316

**Published:** 2023-07-18

**Authors:** Alejandra Bravo, Mario Soberón

**Affiliations:** ^1^ Instituto de Biotecnología Universidad Nacional Autónoma de México Cuernavaca Mexico

## Abstract

Extensive use of chemical insecticides to control insect pests in agriculture has improved yields and production of high‐quality food products. However, chemical insecticides have been shown to be harmful also to beneficial insects and many other organisms like vertebrates. Thus, there is a need to replace those chemical insecticides by other control methods in order to protect the environment. Insect pest pathogens, like bacteria, viruses or fungi, are interesting alternatives for production of microbial‐based insecticides to replace the use of chemical products in agriculture. Organic farming, which does not use chemical pesticides for pest control, relies on integrated pest management techniques and in the use of microbial‐based insecticides for pest control. Microbial‐based insecticides require precise formulation and extensive monitoring of insect pests, since they are highly specific for certain insect pests and in general are more effective for larval young instars. Here, we analyse the possibility of using microbial‐based insecticides to replace chemical pesticides in agricultural production.

## IS THERE A NEED FOR REPLACEMENT OF CHEMICAL PESTICIDES IN AGRICULTURAL PRODUCTION?

Food demand of our increasing human population has been met by agricultural intensification resulting in an important crop production increase (Rezende‐Teixeira et al., 2022). Intensification of agricultural productivity has been achieved by an increase input of fertilizers, herbicides and chemical insecticides, to improve plant growth and to control crop pests, resulting in higher yields and also high quality of food products (Loiseleur, 2017). It has been estimated that pest‐associated losses in agriculture could reach up to 14% (Oerke, 2006). However, in the case of chemical insecticides, it is widely recognized that most of these insecticides have detrimental effects on human health, are toxic to non‐target insects and remain in the environment for long periods of time (Köhler & Triebskorn, 2013). Moreover, evolution of insect resistance to chemical insecticides has resulted in a continuous increase use of these pesticides in order to achieve pest control (Bourguet et al., 2013). In the last century, long‐persistent highly toxic chemical insecticides have been replaced for less‐persistent low‐toxicity insecticides to reduce the negative effects in human health and the environmental problems caused by exposure to these chemicals (Soberón et al., 2023). In addition, different agricultural practices, such as use of genetically modified crops expressing insecticidal proteins, originally found in the bacterium *Bacillus thuringiensis*, have resulted in the reduction of chemical pesticides in agricultural production (Krishna & Qaim, 2012). However, even for these insect‐resistant transgenic crops, the farmers require to use some other insecticide applications for the control of additional secondary pests that are not susceptible to the insecticidal proteins produced in the transgenic plants. Nevertheless, still now day extensive use of chemical pesticides has been shown to be linked to human diseases or affecting non‐target beneficial insects. A recent study on the association between agricultural pesticide usage and cancer incidence among children and adults in 11 states of western United States of America (USA) revealed a strong correlation on the use of fumigants in agriculture, as Metam, and cancer incidence in both children and adults (Joseph et al., 2022). Regarding the negative effects on beneficial insects, it was documented that in March this year, the application of fumigant Fipronil in corn fields of Campeche, México, accompanied by strong winds, caused the death of more than 300,000 honeybees, from around 5000 hives, with a large estimated economic impact on crop productivity and honey production in this region due to the loss of these important pollinators (euro.eseuro.com/local/397813.html). Thus, the replacement of highly toxic chemical pesticides used in agriculture by less toxic compounds is necessary.

## CAN AGRICULTURAL PRODUCTION BE SUSTAINED WITHOUT THE USE OF CHEMICAL PESTICIDES?

Organic agriculture is an agricultural practice that does not rely on the use of inputs like fertilizers, herbicides or chemical pesticides that have adverse effects, depending mainly on integrated pest management practices (IPM) like ecological processes, biodiversity and cycles like crop rotation (Norton & Swinton, 2009). Genetically modified crops are not used in organic farming. The goal of this practice is to prevent pests reaching economic damaging levels while preserving the ecosystem. The organic farming pest control relies on a handful of compounds mainly derived from plants or microorganism. Also, microbial‐based insecticides are used in organic farming for pest control (Olson, 2015; Peshin et al., 2009). Interestingly, microbial‐based insecticides are not harmful to natural enemies of insect pests resulting in high abundance of these natural enemies which contribute for the control of insect pests. However, although organic food demand is continuously increasing, the organic food market represents 6% of the whole market and farmland represents only 1% of the whole farmland in USA (https://fortune.com/2022/09/22/organic‐farming‐popular‐but‐not‐with‐farmers‐converting/).

## CAN MICROBIAL‐BASED INSECTICIDES REPLACE CHEMICAL PESTICIDES IN AGRICULTURAL PRODUCTION?

Insects are susceptible to multiple infectious diseases caused by different microorganisms such as bacteria, fungi or viruses. Microorganisms have some advantage over chemical insecticides such as narrow toxicity to their specific target pests, having no effect on other organisms or beneficial insects and do not persist on the environment. However, some of the advantages of microbial‐based insecticides are seen as burdens by farmers since microorganism‐based insecticides target mainly young larval stages, and take longer time to kill the insect pests, which is seen as a failure. Their narrow specificity is also seen as a problem since additional insecticides may be needed for the control of secondary pests not susceptible to the microbial insecticides. Also, knowledge of their mode of action is important for applying them at the precise conditions and correct timing.

In case of bacterial insect pathogens, *Bacillus thuringiensis* (Bt) has been the most successful bio‐insecticide for the control of different insect pests belonging to lepidoptera, coleoptera or diptera (Pardo‐López et al., 2013). Different Bt strains produce several types of insecticidal proteins mainly during its sporulation phase of growth, which are accumulated in parasporal crystals. The most studied and applied toxins belong to the Cry family of insecticidal proteins. Insecticidal Cry proteins are synthesized as protoxins. To exert their toxic effect, the susceptible larvae must ingest the spore/crystals containing the Cry protoxins, which are then solubilized in the larval gut conditions, where they are processed by larval gut proteases to yield an activated toxin. Cry toxins bind with high affinity to specific larval gut proteins located in the apical membrane of the insect midgut cells. These binding interactions trigger oligomerization and membrane insertion of the toxin into microvilli membranes of the midgut cells. Finally, pore formation by Cry inserted oligomers causes larval gut cell lysis by osmotic shock, causing the death of the larvae (Bravo et al., 2011). Interestingly, most Bt strains produce several insecticidal proteins which broaden their insect specificity and, in some cases, induce synergistic interactions among the different insecticidal proteins enhancing toxicity (Bravo et al., 2011; Pardo‐López et al., 2013). All Bt‐based insecticides are formulated from native Bt strains that produce several Cry toxins. Bt strains were initially characterized by their flagella‐H serotype where strains belonging to serotypes *kurstaki* and *aizawai* have been used to develop insecticides against lepidopteran pests while strains belonging to serotype *tenebrionis* were used for control of coleopteran pests (Couch & Jurat‐Fuentes, 2014). Bt‐based insecticides are used in organic farming and also in intensive agriculture to protect different crops such as cotton, corn, soybean and cruciferous vegetables.

More than 1000 insect viruses have been already described. *Baculovirus* family of viruses account for most of the insecticidal applications. *Baculovirus* have shown narrow specificity for their insect hosts showing no toxicity to other organisms (Fuller et al., 2012; Vasantharaj, 2008). Several hundred species of *Baculovirus* have been characterized from different insect orders such as Lepidoptera, Hymenoptra or Diptera. These virions are enveloped by a nucleocapsid. In the case of *Occlusion*‐derived virus, they are encapsulated by a matrix of either polyhedrin or granulin forming occlusion bodies (OB) which might encapsulate one or several virions. Infection occurs after ingestion of OB by the susceptible larvae. OB are dissolved in the alkaline larval gut conditions releasing the virions which after trespassing the peritrophic membrane of the gut, are able to infect gut cells by entering the cell through interaction with the brush border membrane. Once inside the cell, the nucleocapsids enter the cell nucleus where their replication and transcription of viral genes occur (Haase et al., 2015). Budded virions are produced capable of infecting neighbour gut cells spreading the infection. Finally, *Occlusion*‐derived viruses are assembled in OB where up to 10^10^ virions are produced per larva. OB are resistant to different environmental conditions facilitating their persistence in the environment and further infections improving their spreading and the efficient control of insect pests (Haase et al., 2015). Virus‐based insecticides are commonly used in organic farming but also have been used to manage insect pests that have become resistant to Bt transgenic crops.

Fungal‐based insecticides have been extensively used as a strategy to overcome insect resistance to chemical insecticides. Thus, the fungal‐based insecticides have been applied with sublethal doses of chemical insecticides reducing the evolution of resistance to chemical insecticides (Ambethgar, 2009). *Beauveria bassiana*, *Metarhizium anisopliae*, *Nomuraea rileyi*, *Paecilomyces* spp., *Lecanicillium lecanii* and *Hirsutella thompsonii* have been successfully formulated as insecticides. For infection, fungi hyphae penetrate their host through the cuticle to gain access to the haemolymph, producing toxins and using the haemolymph content as growth substrate spreading infection to the whole body of the insect (Ambethgar, 2009). Fungi are generally formulated as conidia or mycelium that sporulate after application (Behle & Birthisel, 2014). As with BT or virus, fungi have a narrow insect specificity being innocuous to beneficial insects or vertebrates.

To develop commercial insecticides based on microorganism, it is necessary to produce the microorganisms at a large scale and to formulate them to guarantee their storage and application. Different microorganisms have their own challenges for developing efficient formulations. In the case of virus and fungi, maintaining the microorganisms' viability is crucial. This is in contrast to Bt where this is not the case since mixtures of spore/crystals are applied requiring simple formulation ingredients (Behle & Birthisel, 2014).

As mentioned above, the use of microbial‐based insecticides requires extensive monitoring to detect crop damage induced by the pests at early stages, since most microbial‐based insecticides are effective against young larval instars. Technological tools like satellite survey or use of drones to monitor and to apply control tools are now available. As mentioned previously, the use of genetically modified crops is not allowed for organic farming. However, genetically modified crops expressing Bt insecticidal proteins have been shown to be successful in reducing chemical insecticide use in agricultural production (Krishna & Qaim, 2012). Nevertheless, organic farming makes use of IPM techniques allowing the successful control of pests without using chemical insecticides. Thus, combining genetically modified crops with IPM strategies such as extensive monitoring to apply microbial‐based insecticides using precision/patch spraying by using GPS spray maps. IPM strategies such as habitat manipulation, cultural and mechanical control practices, like major ploughing and minor tillage or using additional strategies such as pheromone traps or acoustic pest detection techniques that allow the detecting insects by their species‐specific sounds could be applied along with microbial insecticides (Johnson et al., 2007). In addition, models to predict population dynamics of natural enemies is likely to reduce substantially the use of chemical insecticides in agriculture.

Finally, it is foreseen that tight regulations on the use of chemicals in agriculture and the increasing problem of evolution of insect resistance to these chemicals will increase the use of microbial‐based insecticides for a more environment‐friendly agricultural production.

## AUTHOR CONTRIBUTIONS

**Alejandra Bravo:** Conceptualization (equal). **Mario Soberón:** Conceptualization (equal).

## FUNDING INFORMATION

No funding information provided.

## CONFLICT OF INTEREST STATEMENT

The authors have no conflict of interests.
